# A head-to-tail view of L-selectin and its impact on neutrophil behaviour

**DOI:** 10.1007/s00441-017-2774-x

**Published:** 2018-01-20

**Authors:** Aleksandar Ivetic

**Affiliations:** BHF Centre for Research Excellence, School of Cardiovascular Medicine & Sciences, Faculty of Life Sciences & Medicine, King’s College London, James Black Centre 125, Coldharbour Lane, London, SE5 9NU UK

**Keywords:** Leukocyte trafficking, Ectodomain shedding, Signalling, L-selectin, Neutrophil

## Abstract

L-selectin is a type I transmembrane cell adhesion molecule expressed on most circulating leukocytes, including neutrophils. Engagement of L-selectin with endothelial-derived ligands initiates neutrophil tethering and rolling behaviour along luminal walls of post-capillary venules, constituting the first step of the multi-step adhesion cascade. There is a large body of evidence to suggest that signalling downstream of L-selectin can influence neutrophil behaviour: adhesion, migration and priming. This review will cover aspects of L-selectin form and function and introduce the “triad of L-selectin regulation”, highlighting the inextricable links between adhesion, signalling and ectodomain shedding and also highlighting the cytosolic proteins that interconnect them. Recent advances in how L-selectin impacts priming, transendothelial migration (TEM) and cell polarity will also be discussed.

## L-selectin expression and domain organisation

### Gene expression, domain organisation and glycosylation

**Fig. 1:**
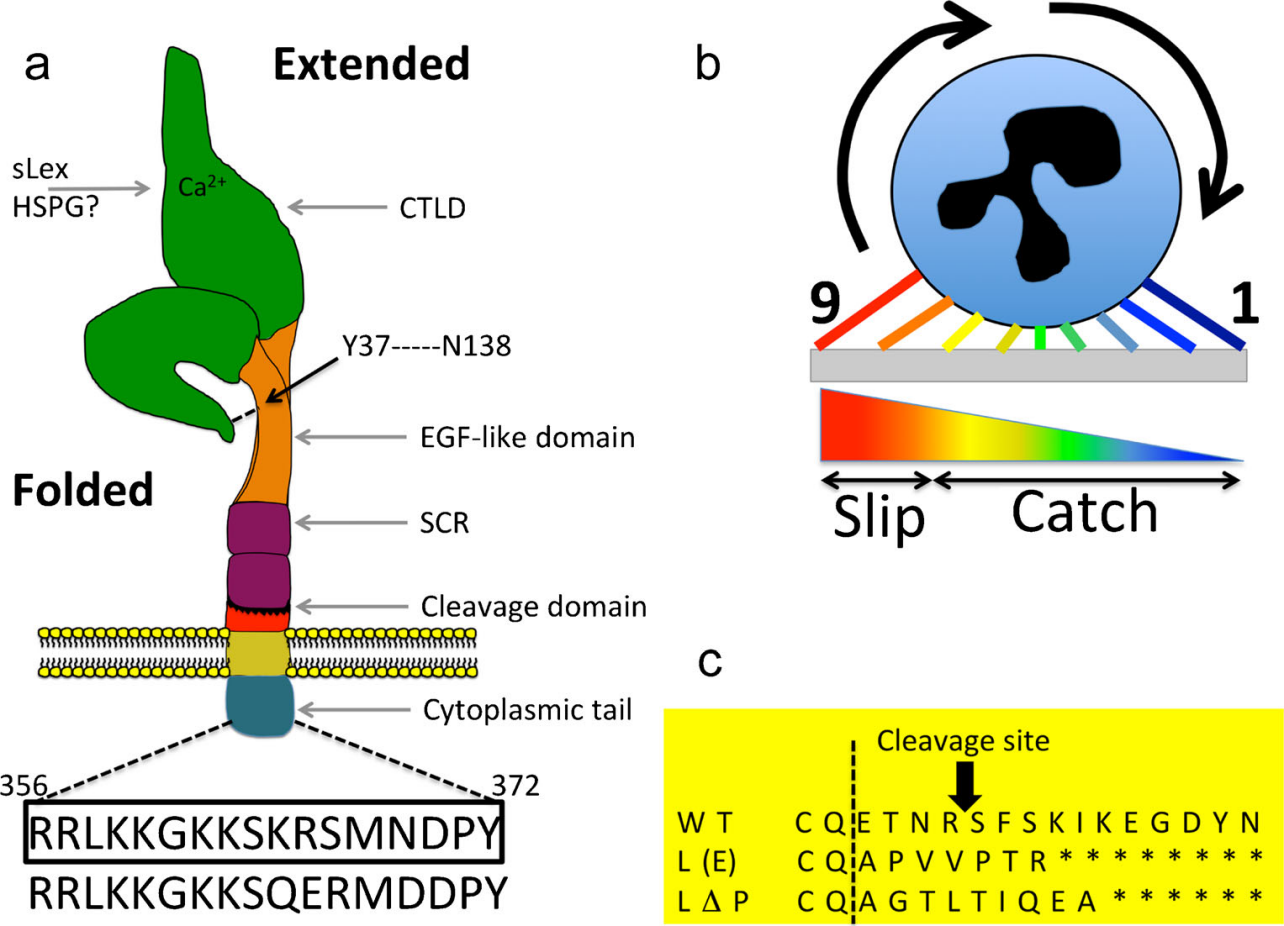
L-selectin form and function. **a** Schematic representation of L-selectin, showing the domain organisation:* CTLD* calcium-type lectin domain;* EGF* epidermal growth factor-like domain;* SCR* sequence consensus repeat; cleavage domain and cytoplasmic tail. Amino acid sequence (356–372) is provided for human (*boxed*) and mouse cytoplasmic tail. Two superimposed impressions of L-selectin are depicted in the folded and extended forms. These drawn forms do not faithfully reflect the crystal structure but are intended to highlight changes in conformation. The folded form is facilitated by a hydrogen bond between tyrosine (*Y*) 37 and asparagine (*N*) 138. Although the Ca2+ binding of the CTLD is essential for sLex interaction, it is currently not clear how HSPGs interact with L-selectin. **b** A schematic depiction of the catch–slip bond that L-selectin experiences during cell rolling. *1* depicts initial L-selectin interaction at the leading edge and *9* is L-selectin released at the rear of the cell, by virtue of the slip bond. The *pseudo-coloured scalene triangle* represents the increase in tensile force (*blue* low tensile force;* red* high tensile force) experienced by L-selectin during rolling, where a transition is made from “catch” to “slip”. **c** Amino acid sequences of the membrane proximal regions of wild-type (*WT*) and sheddase-resistant mouse L-selectin.* Dotted line* represents the boundary between the end of the 2nd SCR and the beginning of the cleavage domain

L-selectin is one of three family members: L-, E- and P-selectin (Ley 2003). Each selectin is defined according to the cell type in which it was first characterised (L = lymphocyte, E = endothelial cell, P = platelet). L-selectin is a type I transmembrane glycoprotein composed of numerous functional and regulatory domains (Ivetic 2013; Wedepohl et al. 2012). All three selectin genes reside in tandem on human chromosome 1, suggesting that an original gene had undergone multiple duplication events during evolution (Watson et al. 1990). Selectin-like genes have been identified in lower organisms and it is not clear if their roles are distinct from mammalian selectins. A P-selectin-like gene has been identified in zebrafish that bears 37–39% identity at the amino acid level to mammalian P-selectin; no other selectin members have yet been identified in zebrafish (Sun et al. 2010). A P-selectin-like gene, *furrowed*, has also been identified in *Drosophila melanogaster*, which localises to epithelial junctions and regulates planar cell polarity (Chin and Mlodzik 2013). Mutations within *furrowed* leads to developmental defects in the eye and mechanosensory bristles (Leshko-Lindsay and Corces 1997). L-selectin is also expressed in developing trophoblasts (Feng et al. 2017), sertoli cells (Freeman et al. 2002) and skeletal muscle stem cells (Torrente et al. 2003). While adhesion plays a fundamental role in the function of these extra-immune events, little has been followed up on these findings. Each selectin possesses an N-terminal calcium-dependent (C-type) lectin domain (CTLD), an epidermal growth factor (EGF)-like domain, a varying number of short complement-like repeat (SCR) domains, a transmembrane domain and a short cytoplasmic tail (see Fig. 1a). The predicted molecular weight of L-selectin is approximately 30 kDa but the actual molecular weight ranges between 70 and 100 kDa and appears to be cell type-specific. These findings suggest that differential N- and O-linked glycosylation of L-selectin could impact its form and function on different immune cell subsets, as well as interaction with other molecules in *cis* (on the same plasma membrane) and *trans* (between different cells).

### The C-type lectin domain (CTLD) and epidermal growth factor (EGF)-like domain

The CTLD binds to glycans that decorate proteins or lipids and are typically presented by endothelial cells or other leukocytes. The minimal structural determinant for a selectin ligand is composed of a branched tetrasaccharide, called sialyl Lewis x (sLe^x^), containing: sialic acid, galactose, fucose and N-acetyl glucosamine (expressed as: Siaα2,3Galβ1,4 (Fucα1,3)GlcNAc) (McEver et al. 1995). L-selectin can bind to sulfated variants of sLe^x^ with higher affinity, the expression of which appears to be tissue-specific. For example, high endothelial cells lining venules entering peripheral lymph nodes constitutively express sulfo-sLe^x^ (Bistrup et al. 1999). Intriguingly, L-selectin on human neutrophils is itself decorated with sLe^x^ and previous studies have shown that it can act as a ligand for E-selectin (Zollner et al. 1997). This human-specific glycan modification suggests that mechanisms mediating initial recruitment (i.e., tethering and rolling) could be species-specific. Of note, sLe^x^ is predominantly N-linked to L-selectin, whereas the archetypal selectin ligand, P-selectin glycoprotein ligand-1 (PSGL-1), is typically O-linked (Buffone et al. 2013; Mondal et al. 2013). Moreover, studies using the glycomimetic Rivipansel, which selectively masks E-selectin recognition of sLex on L-selectin, reveals an important role for L-selectin in transitioning neutrophils from rolling to arrest (see “L-selectin-dependent signalling: homo/heterotypic L-selectin clustering”). This has led to a paradigm shift in understanding how mechanosignalling is transduced in mouse and human neutrophils during recruitment (Morikis et al. 2017).

Just over 20 years ago, L-selectin was first shown to act as a shear-dependent cell adhesion molecule (Finger et al. 1996). L-selectin undergoes sub-second changes in bond lifetime with its ligand under flow conditions, categorised into “catch” and “slip” bonds (see Fig. 1b). Initial contact between the CTLD and ligand exerts a low tensile strength, which starts at the leading edge of the cell. Under optimal shear stress conditions (0.3–1.0 dyne per cm^2^), the tensile force between the CTLD and its ligand increases to unfold and expose a greater region for ligand binding. At this point, ligand interaction is positioned beneath the rolling cell. The bond lifetime increases under this condition and is known as the “catch” bond. As time proceeds, the tensile force between the CTLD and ligand increases further as the leukocyte rolls over the initial site of contact, where the bond is now at the trailing end of the cell. As the tensile force exceeds the limit for catch bonds, the bond lifetime decreases and “slips” to release the CTLD from its ligand. Under conditions of abundant ligand availability, a new catch bond will form at the new leading edge to allow the process repeat, culminating in classic cell rolling behaviour. X-ray crystal structures of the L-selectin CTLD and EGF-like domains have recently been solved (Mehta-D'souza et al. 2017; Wedepohl et al. 2017). An “open” to “closed” allosteric conversion of a seven amino acid loop (containing residues 83–89) within the CTLD, which interacts with Ca^2+^ and sLe^x^, is thought to underlie the catch bond mechanism (Mehta-D'souza et al. 2017). Structural comparison with family members E- and P-selectin reveals highly conserved features, again implying conserved mechanisms underlying ligand binding. The CTLD and the EGF-like domain are connected by a flexible hinge region that contributes to selectivity and strength of ligand binding under flow conditions (Lou et al. 2006). Aside from adhesion under flow, others have shown that L-selectin can bind to negatively charged (heavily sulfated) glycosaminoglycans (GAGs) (Kawashima et al. 2000; Kawashima et al. 1999; Kawashima et al. 2003; Kitaya and Yasuo 2009). There are several types of GAGs, which include: heparan sulfate, chondroitin sulfate, dermatan sulfate and keratan sulfate. While these GAGs reside on the apical aspect of endothelial cells, they appear to be more concentrated within the basolateral aspect of the endothelium (Celie et al. 2009; Rzeniewicz et al. 2015; Stoler-Barak et al. 2014). In silico studies have identified a positively charged “patch” on the CTLD that can bind to GAGs in a pH-dependent manner (Martinez et al. 2013), although this has not been confirmed experimentally.

### The SCR domain

The SCR bears homology with complement regulatory proteins, alternatively named: sushi domains, regulators of complement activation or complement control proteins. Two conserved disulfide bonds provide the secondary structure of a single SCR domain. Each selectin family member possesses a varying number of SCRs. Each domain acts as spacer module between the CTLD and the plasma membrane, allowing the selectins to stand head and shoulders above other cell adhesion molecules, which is essential for successful tethering, particularly in postcapillary venules greater than 20 μm in diameter (Stein et al. 1999; von Andrian et al. 1995). L-selectin only possesses two SCRs, whereas P-selectin possesses nine (Ley 2003). This disparity is likely due to the anchorage of L-selectin to microvilli, placing the molecule in an already advantageous subcellular location for tethering under flow conditions.

### The cleavage domain and cytoplasmic tail of L-selectin

Both of these domains are discussed in greater detail in “Ectodomain shedding of L-selectin” and “The cytoplasmic tail of L-selectin: a central regulator of adhesion, signalling and ectodomain shedding”.

## Ectodomain shedding of L-selectin

Unlike its family members, L-selectin possesses a unique membrane-proximal cleavage site positioned nine amino acids up from the plasma membrane (Kahn et al. 1994; Migaki et al. 1995) (see Fig. 1a, c). Ectodomain shedding of L-selectin from neutrophils is triggered by numerous extracellular cues, such as high-density ligand-induced clustering of L-selectin (Liu and Kiick 2011), CD18 integrin clustering (Walzog et al. 1994), exposure to oxidised LDL (Lehr et al. 1995), osmotic stress (Rizoli et al. 1999) and numerous pro-inflammatory stimuli (Haribabu et al. 1997; Jutila et al. 1989; Kishimoto et al. 1989; Smalley and Ley 2005). Ultimately, ectodomain shedding serves to rapidly shut down L-selectin-dependent adhesion and signalling. L-selectin is turned over at the plasma membrane at steady state in leukocytes, including neutrophils (Gomez-Gaviro et al. 2000; Zhao et al. 2001). Significant decreases in the surface expression of L-selectin has been observed in ageing neutrophils, which inversely correlates with increased CXCR4 expression, a bone marrow-homing receptor that is required for neutrophil clearance by bone marrow-resident macrophages (Casanova-Acebes et al. 2013; Zhang et al. 2015).

### Inducible ectodomain shedding of L-selectin

Neutrophil activation with pro-inflammatory stimuli such as formyl peptides (from Gram-negative bacteria or host cell-derived mitochondria), TNF or Toll-like receptor agonists leads to robust and rapid shedding of L-selectin within minutes (Hazeldine et al. 2015; Killock and Ivetic 2010). Indeed, loss of L-selectin expression in neutrophils is used as the gold standard to assess neutrophil activation in vivo and in vitro. Reduced L-selectin expression is inversely correlated with increased Mac-1 (αMβ2, or CD11b/CD18) integrin expression (Kishimoto et al. 1989). Mac-1 is typically associated with numerous neutrophil effector functions, such as intraluminal crawling, TEM (Phillipson et al. 2006; Sumagin et al. 2010), phagocytosis (Thompson et al. 1984) and chemotaxis (Heit et al. 2005). L-selectin shedding leads to the release of a soluble bioactive ectodomain, which can bind ligands expressed in the vasculature (Schleiffenbaum et al. 1992). In this context, soluble (s)L-selectin does not conform to the catch–slip bond paradigm but acts as a potent competitor of cell-associated L-selectin. Numerous reports suggest sL-selectin can either be protective (Albertini et al. 1999; Haught et al. 1996; Patiar et al. 2002) or a risk factor in certain clinical settings (Atalar et al. 2001; Signorelli et al. 2003; Siminiak et al. 1998, 1999; Wei et al. 2011), implying complex roles in disease pathogenesis. The amount of sL-selectin measured from the serum of healthy individuals ranges from 0.7 to 1.5 μg per mL, which can rise to 2–3 μg per mL in patients with autoimmune diseases such as Lupus (Font et al. 2000) or primary Sjögren’s syndrome (Garcia-Carrasco et al. 2000). Studies in mice have shown the half-life of sL-selectin can be up to 20 h (Tu et al. 2002). While sL-selectin in healthy individuals is thought to regulate homeostatic leukocyte trafficking, the masking of ligands through acute rises in sL-selectin production can negatively impact on adhesion during the inflammatory response. For example, sL-selectin released from neutrophils responding to acute pain can self-limit recruitment and therefore lessen the extent of inflammation in that area (Strausbaugh et al. 1999). The major source of sL-selectin in human serum is not known, although mice expressing wild-type (WT) L-selectin exclusively on T-cells contribute up to 70% of total sL-selectin (Galkina et al. 2003), suggesting a significant contribution of sL-selectin originates from this cell type. Given the disparity in the percentage of circulating lymphocytes and neutrophils in mice and humans (lymphocytes = 90% in mice vs .50% in humans; neutrophils = 25% in mice vs. 70% in humans), the source of sL-selectin and its contribution to (patho)physiology may differ between species.

### L-selectin shedding during adhesion and migration

L-selectin shedding can be activated during rolling and TEM under flow conditions (Lee et al. 2007; Rzeniewicz et al. 2015). For example, in parallel plate flow chamber studies, primary human neutrophils engaged in prolonged rolling activity on immobilised sLe^x^ can over time re-enter flow. Labelling of primary human neutrophils with fluorescently conjugated anti-L-selectin antibody enabled visualisation of cleaved L-selectin, deposited as fluorescent tracks along rolling contact sites. Furthermore, sustained release of L-selectin led to faster rolling speeds that eventually culminated in detachment of the neutrophil back into flow. The term “mechanical shedding of L-selectin” was coined to explain this phenomenon, which could be blocked specifically with synthetic inhibitors of p38 MAPK, implying that intracellular signalling is underpinning the mechanism (Lee et al. 2007). Although this observation stems from in vitro studies, it has yet to be described and characterised in vivo (see “The importance of mechanosignalling in circulating neutrophils”). Earlier studies showed, under “static” conditions (i.e., in the absence of haemodynamic shear stress), a direct correlation between neutrophil TEM and L-selectin expression. Transmigrated neutrophils harvested from beneath activated endothelial monolayers registered L-selectin-negative by flow cytometry, suggesting that L-selectin shedding occurred during recruitment and/or during TEM (Allport et al. 1997). Integrating fluorescence timelapse microscopy with the parallel plate flow chamber has exposed exactly where and when L-selectin shedding occurs in transmigrating leukocytes (Rzeniewicz et al. 2015) (see “L-selectin: a driver of invasion and cell polarity”).

### Genetic and pharmacologic approaches towards blocking L-selectin shedding

#### L-selectin sheddases

There is abundant evidence to suggest that the enzyme responsible for cleaving L-selectin on neutrophils is a disintegrin and metalloproteinase 17 (ADAM17) or TNF-alpha converting enzyme (Ager 2012; Condon et al. 2001; Long et al. 2010; Peschon et al. 1998; Tang et al. 2011). Other members, such as ADAM8 and ADAM10, can also cleave L-selectin (Gomez-Gaviro et al. 2007; Le Gall et al. 2009). ADAM8 is expressed at the plasma membrane and within intracellular (“specific” and “small storage”) granules of neutrophils. Soluble ADAM8 can be enzymatically released from the neutrophil plasma membrane but the enzyme mediating this event is not known. There is some evidence to suggest that ADAM8 can autoactivate in vitro using a human epithelial kidney cell line but this has not been seen in primary neutrophils. ADAM8 is highly abundant and enzymatically active in the synovia of human rheumatoid arthritic joints. Soluble ADAM8 can cleave cell-associated L-selectin, suggesting that, unlike ADAM17, it can cleave L-selectin in *trans* (Gomez-Gaviro et al. 2007). Cell-associated ADAM10 has also been shown to cleave other substrates in *trans*, suggesting that L-selectin shedding (for example during TEM) may be cleaved by endothelial-derived ADAM10.

#### Regulation of ADAM17 activity

ADAM17 is expressed and stored in vesicles in numerous leukocyte subtypes and can be rapidly mobilised to the plasma membrane in response to cell-activating stimuli (Ebsen et al. 2013; Killock and Ivetic 2010). ADAM17 knockout mice are embryonic lethal (Peschon et al. 1998) and therefore the transfer of ADAM17 foetal liver cells (E15.5) into lethally irradiated WT recipient (or “chimeric”) mice is a typical approach to study the in vivo role of ADAM17-deficient haematopoietic cells. Flow cytometry reveals that basal turnover of L-selectin is blocked in ADAM17-null chimeric mice. Therefore, ADAM17-null neutrophils express significantly higher levels of surface L-selectin than WT neutrophils, a feature that is mirrored when the L-selectin cleavage site is mutated in vivo (see “Cleavage-resistant mutants of L-selectin and sheddase inhibitors”). Also, L-selectin expression remains unchanged in neutrophils harvested from peritoneal cavities of ADAM17-null mice challenged with *E. coli* or thioglycollate (Long et al. 2012; Tang et al. 2011). Given that these studies were conducted in chimeric mice, this reaffirms that ADAM17 cannot cleave L-selectin in *trans*. ADAM17 is therefore the dominant “sheddase” in cleaving L-selectin on neutrophils. However, given that ADAM8 is released as a soluble enzyme by sheddase activity, it is still not clear if potential cascades of sheddase activity are required for the proteolytic release of L-selectin in some (patho)physiological settings. In leukocytes, ADAM17 activity is controlled by two major kinases: PKC and p38 MAPK. Systematic activation of either PKC or p38 MAPK reveals fundamental differences in ADAM17-dependent shedding of L-selectin (Killock and Ivetic 2010). PKC-induced shedding of L-selectin is strongly dependent on regulatory elements within the cytoplasmic tail of L-selectin (e.g., serine phosphorylation and Ezrin-Radixin-Moesin binding; see “Serine and tyrosine phosphorylation of the L-selectin tail” and “Ezrin–Radixin–Moesin (ERM)”), whereas p38 MAPK-induced shedding of L-selectin is independent of these regulatory elements. In contrast, threonine phosphorylation of the ADAM17 cytoplasmic tail is an essential pre-requisite for p38 MAPK-induced shedding, which is not required for PKC-induced shedding. Tipping intracellular signalling predominantly towards PKC or p38 MAPK activation will very much depend on the origin of the input signal. Indeed, fMLP/LPS/TNF-induced shedding of L-selectin is mediated more through p38 MAPK than PKC (Fan and Derynck 1999; Killock and Ivetic 2010). In contrast, shedding of L-selectin induced by T-cell receptor signalling is driven predominantly by PKCα (Gharbi et al. 2013).

Non-steroidal anti-inflammatory drugs (NSAIDs) can promote the shedding of L-selectin in neutrophils (Diaz-Gonzalez et al. 1995). While this mode of action is one of many anti-inflammatory effects of NSAIDs, it is not thought to involve classic inhibition of cyclooxygenase and prostaglandin production. Instead, two theories have been put forward: the first is that NSAIDs can directly block the binding of calmodulin to the cytoplasmic tail of L-selectin (Cantabrana et al. 1995), where calmodulin dissociation typically leads to L-selectin shedding (more detail of L-selectin/calmodulin interaction is provided in “Calmodulin (CaM) interaction with the L-selectin tail”). The second theory relates to NSAIDs reducing intracellular ATP in neutrophils and its positive correlation with L-selectin shedding (Gomez-Gaviro et al. 2000). Acute depletion of cellular ATP levels in neutrophils, using sodium azide to block mitochondrial respiration, promotes a similar effect. Interestingly, however, while extracellular ATP can promote L-selectin shedding in lymphocytes (Jamieson et al. 1996), it cannot in neutrophils (Sengstake et al. 2006). Neutrophils can secrete ATP (Chen et al. 2006b; Eltzschig et al. 2006) and while this reduces net intracellular levels, the shedding of L-selectin is likely not driven via cell surface ATP or adenosine receptors (e.g., A3, P2X7R and P2Y2) (Barletta et al. 2012). More recently, the mechanism of action of NSAIDs driving L-selectin shedding was postulated to occur via the production of superoxide (Dominguez-Luis et al. 2013). Furthermore, oxidation of critical cysteine residues within the ectodomain of ADAM17 enhances its catalytic activity for L-selectin shedding (Wang et al. 2009). These examples of intracellular and extracellular regulatory mechanisms of ADAM17 activity are clearly complex and their physiological significance in health/disease is yet to be addressed.

#### Cleavage-resistant mutants of L-selectin and sheddase inhibitors

Understanding the contribution of L-selectin shedding to leukocyte behaviour has been achieved mainly through pharmacologic and genetic approaches. Cell lines that do not express endogenous L-selectin have been used to cleanly investigate the contribution of L-selectin in its WT or non-cleavable form. An eight amino acid deletion (amino acids: MIKEGDYN, termed “ΔM-N”) of human L-selectin renders the protein non-cleavable or “sheddase-resistant” (Chen et al. 1995). Two different mouse models have been engineered, each expressing a domain swap mutation between the L-selectin cleavage site and the corresponding region of E or P-selectin, called: “L(E)” and “LΔP” (Galkina et al. 2003; Venturi et al. 2003) (see Fig. 1c). The L(E) model is a global knock-in mutation, while the LΔP model expresses sheddase-resistant L-selectin from a transgene under the control of a CD2 promoter (T-cell lineage-specific). Importantly, the LΔP transgene is expressed in L-selectin-deficient mice giving rise to L-selectin expression only in T-lymphocytes. Both in vivo models exhibit delayed leukocyte emigration across venular walls, suggesting the importance of L-selectin shedding in transmigration. Blocking L-selectin shedding in primary human neutrophils does not affect TEM rates across activated endothelial monolayers in vitro, implying that the delayed emigration phenotype seen in vivo could be due to a delay in breaching other physical barriers beyond the endothelium, e.g., the basement membrane or pericytes (Alon and Nourshargh 2013; Nourshargh and Alon 2014; Proebstl et al. 2012). To date, detailed examination of the migratory behaviour of sheddase-resistant interstitial neutrophils has not been undertaken. However, bright-field intravital microscopy reveals that L(E) neutrophils emigrating in response to localised keratinocyte-derived chemokine (KC or CXCL1) gradients remain close to the vessel wall (Venturi et al. 2003). Given that this phenotype is copied in L-selectin-null neutrophils (Hickey et al. 2000), it begs the question of whether the membrane-retained fragment (MRF; the by-product of L-selectin shedding) plays a more direct role in chemotaxis, as the MRF is absent in both sheddase-resistant (L(E)/LΔP) and L-selectin-null neutrophils.

Studies where L-selectin cannot be genetically manipulated (e.g., in primary human neutrophils) have relied on the use of hydroxamate-based synthetic inhibitors of ADAM17 (Ro-31-9790, KD-IX-73-3, TAPI-0, TAPI-1, TMI005 and GM6001) being the most commonly used/cited. While these inhibitors are far from specific to ADAM17, most have demonstrated retention of L-selectin expression following cellular activation and some studies have shown increased accumulation of neutrophils along inflamed postcapillary venules in vivo (Hafezi-Moghadam et al. 2001). The recent development and refinement of an anti-human ADAM17 phage display antibody, targeting the active site, should provide clearer understanding of the importance of ADAM17-dependent shedding of L-selectin in human neutrophils (Tape et al. 2011).

## The cytoplasmic tail of L-selectin: a central regulator of adhesion, signalling and ectodomain shedding

The cytoplasmic tails of selectin family members bear little resemblance to one another, suggesting unique contributions to intracellular signalling (Ivetic and Ridley 2004b; Ley 2003). Clustering human L-selectin with monoclonal antibody or exposure to physiological ligand can promote tyrosine phosphorylation on intracellular proteins that include MAP kinases (Waddell et al. 1995), strongly implying that L-selectin can transduce intracellular signals. Similar methods used to cluster L-selectin can also activate β1 (Giblin et al. 1997) and β2 integrins (Green et al. 2004; Hwang et al. 1996), promoting the respective adhesion to fibronectin/vascular cell adhesion molecule-1 (VCAM-1) and intercellular adhesion molecule-1 (ICAM-1). Clustering of L-selectin also increases chemokine receptor expression in lymphocytes, which in turn increases efficiency in chemotaxis (Ding et al. 2003; Duchesneau et al. 2007; Subramanian et al. 2012). Many of these observations were made before any intracellular binding partners for L-selectin were identified and characterised. Needless to say, the L-selectin tail is likely to play a crucial role in many if not all of the cellular responses following L-selectin clustering (see Table 1 for examples). Therefore, the identity and nature of the binding partner provides invaluable insight into how signals are potentially propagated downstream of L-selectin-dependent adhesion leading to changes in cell behaviour. The L-selectin tail is composed of 17 amino acids and is highly basic, possessing a theoretical isoelectric point (pI) of 11.17 (for human L-selectin). Despite its small size, numerous binding partners of the L-selectin tail have been identified: alpha-actinin, calmodulin (CaM), Ezrin-Radixin-Moesin (ERM), PKC isozymes and, most recently, μ1 alpha-adaptin. Other putative binding partners co-precipitate in anti-L-selectin immunoprecipitates but have not been validated as direct binding partners (see “Other indirect binding partners of the L-selectin tail”). The diverse nature in binding partners likely reflects their unique contributions to adhesion, signalling and shedding of L-selectin.

**Table 1:** The impact of clustering L-selectin with ligand or anti-L-selectin antibody on cell behaviour/function

Cell type and organism	Treatment	Cellular outcome	Reference
Human PMN	AMC	Clustering of L-selectin by different anti-L-selectin (DREG) antibodies promotes ectodomain shedding.	(Palecanda et al. 1992)
Human PMN	Sulfatides	Increases intracellular calcium and enhances expression of TNF-α and IL-8 mRNA in neutrophils.	(Laudanna et al. 1994)
Human PMN	AMC	Potentiates oxidative burst in neutrophils.	(Waddell et al. 1994)
Human PMN	AMC	Increases in H_2_O_2_ and intracellular calcium.	(Crockett-Torabi et al. 1995)
Human PMN	AMC	Enhancement of tyrosine phosphorylation and activation of MAP kinase.	(Waddell et al. 1995)
Human PMN	AMC	Increases adhesive function of Mac-1 (CD11b/CD18) β2-integrin.	(Simon et al. 1995)
Human PMN	Sulfatides	Engagement of L-selectin impairs the actin polymerising capacity of β2-integrins on neutrophils	(Ng-Sikorski et al. 1996)
Canine PMN	AMC	L-selectin stimulation of canine neutrophil initiates calcium signalling, secondary to tyrosine kinase activation.	(Crockett-Torabi and Fantone 1997)
Human, mouse and rat leukocytes	AMC and glycomimetics	Ligation of L-selectin through conserved regions within the lectin domain activates signal transduction pathways and integrin function in human, mouse and rat leukocytes.	(Steeber et al. 1997)
Human PMN	AMC and co-stimulation with chemoattractants	Synergy between L-selectin signalling and chemotactic activation during neutrophil adhesion and transmigration.	(Tsang et al. 1997)
Human PMN	Sulfatides	Activation of p21-Activated Kinases (Paks), possibly via L-selectin.	(Huang et al. 1998)
Human PMN	AMC	Alterations in cell rigidity, the cytoskeleton and co-localisation with CD18.	(Simon et al. 1999)
Human PMN	AMC	L-selectin signalling of neutrophil adhesion and degranulation involves p38 mitogen-activated protein kinase.	(Smolen et al. 2000)
Human PMN	AMC	Evidence for a signalling partnership between urokinase receptors (CD87) and L-selectin (CD62L) in neutrophils.	(Sitrin et al. 2001)
Human PMN	AMC by microspheres	Size and frequency of receptor clustering modulates L-selectin-dependent signalling via p38 MAPK and ERK/MEK in neutrophils.	(Green et al. 2003)
Human PMN	Challenge with soluble E-selectin	Shear-dependent capping of L-selectin and P-selectin glycoprotein ligand 1 by E-selectin signals activation of high-avidity beta2-integrin on neutrophils.	(Green et al. 2004)
Human PMN	AMC	c-Abl is involved in the F-actin assembly triggered by L-selectin cross-linking.	(Chen et al. 2006a)
Human and mouse leukocytes	Sulfatide and AMC	Up-regulation of leukocyte CXCR4.	(Duchesneau et al. 2007)
Human PMN	Challenge with E-selectin	Neutrophil adhesion to E-selectin under shear promotes the redistribution and co-clustering of ADAM17 and L-selectin.	(Schaff et al. 2008)
Mouse PMN	Clustering of PSGL-1 and L-selectin	The PSGL-1-L-selectin signalling complex regulates neutrophil adhesion under flow.	(Stadtmann et al. 2013)
Human PMN	E-selectin-dependent adhesion	Selectin catch-bonds mechanotransduce integrin activation and neutrophil arrest on inflamed endothelium under shear flow.	(Morikis et al. 2017)
Jurkat T-cells	IL-2 challenge	Regulation of L-selectin mRNA in Jurkat cells. Opposing influences of calcium and protein kinase C-dependent signalling pathways.	(Kaldjian and Stoolman 1995)
Human lymphocytes	L-selectin binding to Glycam-1	GlyCAM-1, a physiologic ligand for L-selectin, activates beta 2 integrins on naive peripheral lymphocytes.	(Hwang et al. 1996)
Human T-cells	AMC	L-selectin cross-linking induces integrin-dependent adhesion: evidence for a signalling pathway involving tyrosine kinases but not PKC.	(Sikorski et al. 1996)
Human Jurkat T-cells	AMC and glycomimetics	L-selectin activates the Ras pathway via the tyrosine kinase p56lck.	(Brenner et al. 1996)
Jurkat T-cells	AMC and glycomimetics	L-selectin activates JNK via src-like tyrosine kinases and the small G-protein Rac.	(Brenner et al. 1997)
Jurkat T-cells	AMC and glycomimetics	L-selectin stimulates the neutral sphingomyelinase and induces release of ceramide.	(Brenner et al. 1998)
Human lymphocytes (primary and cell lines)	AMC	Intracellular mechanisms of L-selectin-induced capping.	(Junge et al. 1999)
Jurkat T-cells	AMC after surfactant challenge	Surfactant modulates intracellular signalling of the adhesion receptor L-selectin.	(Brenner et al. 2000)
Jurkat T-cells	AMC	Mechanisms of L-selectin-induced activation of the nuclear factor of activated T-lymphocytes (NFAT).	(Brenner et al. 2002)
Mouse T-cells	Challenge cells expressing L-selectin with AgC10	AgC10 binding to L-selectin inhibits IL-2 secretion and T cell proliferation.	(Alcaide and Fresno 2004)
Mouse naïve T-cells	Antibody-mediated clustering of CD3 and L-selectin	Co-stimulation of T-cell proliferation by anti-L-selectin antibody is associated with the reduction of a cdk inhibitor p27.	(Nishijima et al. 2005)
Jurkat T-cells	AMC	Activation of c-Abl and phosphorylation of the terminal tyrosine residue in the L-selectin tail.	(Chen et al. 2007)
Jurkat T-cells	AMC	L-selectin ligation-induced CSF-1 gene transcription is regulated by AP-1 in a c-Abl kinase-dependent manner.	(Chen et al. 2008)
Jurkat T-cells	Sulfatide exposure	Critical role of Lck in L-selectin signalling induced by sulfatides engagement (direct interaction not confirmed).	(Xu et al. 2008)
Mouse splenocytes and lymphocytes	AMC with primary antibody alone	L-selectin and CCR7 synergise to promote increased chemokine responsiveness for T-cell homing	(Subramanian et al. 2012)
Human PBMCs and lymphoma cell line	AMC	The L-selectin antibody FMC46 mediates rapid, transient increase in intracellular calcium in human PBMCs and Daudi lymphoma cells.	(Po et al. 1995)
Human PBMC	AMC	L-selectin clustering induces association of tyrosine–phosphorylated Cbl with CrkL and Grb2 (direct interaction not confirmed)	(Brenner et al. 2001)
Human Monocytes	AMC and adhesion to sLe^x^	Ligand-induced clustering of L-selectin promotes CaM and ERM from neighbouring tails.	(Killock et al. 2009)
Monocytes and macrophages	Glycodelin-A challenge	Glycodelin-A interacts with L-selectin to induce IL-6 production in monocytes/macrophages by activating the ERK signalling pathway	(Lee et al. 2012)

The table provides a chronological overview of cellular outcomes following L-selectin clustering in neutrophils, monocytes and lymphocytes. These experimental approaches have enabled researchers to understand the signalling potential of L-selectin. Note that most of these procedures were conducted on cell suspensions, so it is not fully understood whether L-selectin can influence similar cellular outcomes in isolation in vivo

*PMN* neutrophil, *AMC* antibody-mediated clustering of L-selectin, *PBMC* peripheral blood mononuclear cells

### Serine and tyrosine phosphorylation of the L-selectin tail

The tail of human L-selectin contains two serine residues at positions 364 (S364) and 367 (S367) and a single tyrosine at position 372 (Y372) (Fig. 1a). The cytoplasmic tails of mouse and human L-selectin carry 82% identity at the amino acid level and are 100% identical within the first 10 membrane-proximal amino acids. S364 is common to both mouse and human L-selectin, suggesting conserved mechanisms in phosphorylation at this site between species. Y372 is the last amino acid on the L-selectin tail. Although one study has demonstrated Y372 phosphorylation in response to antibody-mediated clustering of L-selectin (Brenner et al. 1997), its contribution to signalling or adhesion is not fully understood. S364 and S367 are phosphorylated in response to fMLP or chemokine receptor stimulation (Haribabu et al. 1997). Cells expressing L-selectin with alanine mutations at S364 and S367 revealed no phosphorylation of Y372 when challenged with a panel of potent neutrophil chemoattractants (Haribabu et al. 1997). It could be that Y372 is phosphorylated only in response to outside–in signalling, whereas S364/S367 phosphorylation occurs exclusively in response to inside–out signalling. Given the spacing between S364 and S367 and assuming the L-selectin tail adopts an alpha helix in its native form, it is likely that these serine residues are positioned on opposite faces of the tail. Biophysical experiments have shown that the tail of L-selectin has the potential to interact with highly negatively charged phosphatidylserines enriched within the inner leaflet of the plasma membrane (Deng et al. 2011). It has been postulated that phosphorylation of S367 promotes L-selectin repulsion from the negatively charged phospholipids within the inner leaflet of the plasma membrane. Mouse L-selectin lacks S367 but contains an extra aspartate (D) residue at position 369. It is possible that a negative charge cloud at D369 (see Fig. 1a for mouse L-selectin tail sequence) is sufficient to prevent L-selectin from interacting with the inner leaflet of the plasma membrane, which could render the molecule constitutively “peeled off” from the inner leaflet. Both PKC theta and iota can bind to the non-phosphorylated tail of human L-selectin and catalyse the phosphorylation of S364 and S367 (Kilian et al. 2004). Once phosphorylated, PKCα can then bind the L-selectin tail from where it is thought to mediate signalling events through serine/threonine phosphorylation of nearby signalling substrates or other receptors. What is not clear from this study is whether PKCα is binding to the MRF or the full-length form of L-selectin. More recent insight into T-cell receptor signalling would suggest PKCα mediates binding to the MRF of L-selectin (Gharbi et al. 2013).

## Cytosolic binding partners of L-selectin

### Calmodulin (CaM) interaction with the L-selectin tail

The cytoplasmic tail of L-selectin is known to play a crucial role in regulating the shedding of L-selectin. Monoclonal (CA21) and polyclonal (JK924) antibodies, respectively targeting the cytoplasmic and cleavage domains of L-selectin, were used in immunoprecipitation experiments and identified CaM as a novel binding partner (Kahn et al. 1998). The binding was shown to occur specifically with purified CaM and peptide corresponding to the L-selectin tail (Matala et al. 2001). CaM is an 18-kDa ubiquitous calcium-binding protein that can bind to and regulate a multitude of different protein targets, thereby affecting many different cellular functions. CaM consists of two structurally related globular domains located at the N- and C-termini, where each can bind two calcium ions. CaM acts as a negative regulator of shedding and its constitutive association with the L-selectin tail in resting cells, imposes a conformational constraint on the cleavage site that renders it resistant to proteolytic attack by ADAM17 (Kahn et al. 1998). Biophysical and in silico assessments of L-selectin/CaM interaction reveal that CaM binds two regions on L-selectin, one within the cytoplasmic tail and another within the transmembrane domain (Gifford et al. 2012). By binding to both domains, CaM is postulated to “pull” the L-selectin cleavage site down towards the plasma membrane. This ratchet-like activity is thought to underlie why ADAM17 cannot access the L-selectin cleavage site when CaM is bound. Currently, it is unclear if the hydrophobic transmembrane domain is pulled into the cytosol by CaM or how the hydrophilic membrane-proximal extracellular domain would become the de facto transmembrane domain. Co-precipitation of CaM in anti-L-selectin immunoprecipitates is calcium-dependent (Matala et al. 2001); however, CaM can bind to the L-selectin tail in a calcium-independent manner (Killock et al. 2009). These observations suggest multiple routes towards interaction.

An alternative mode of CaM binding to the L-selectin tail comes from in silico approaches. Using a pre-existing NMR structure of an extended conformation of CaM, the C-terminal globular domain of CaM is proposed to interact with L-selectin, allowing the free N-terminal domain to interact with other potential binding partners, such as K-Ras. Interestingly, K-Ras is enriched in microvilli of leukocytes (Hao et al. 2008) and CaM/K-Ras co-localise at the plasma membrane of living cells (Villalonga et al. 2001). These observations might shed light on how specific clustering of L-selectin is proposed to signal to Ras in T-cells (Brenner et al. 1996). A more recent study has shown that the Unique domain of c-Src (common to all Src family kinases; SFKs) can interact with CaM in cells, further indicating how some of the observed signal transduction events involving SFKs could be mediated (Perez et al. 2013). Clearly, more work is required to determine if CaM adopts a folded or extended conformation (and if calcium binding is necessary) to regulate L-selectin-dependent adhesion, shedding and signalling in neutrophils. Recent biochemical and cell biological data show that phosphorylation of S364 but not S367, in human L-selectin is essential for CaM dissociation (Rzeniewicz et al. 2015).

### Ezrin–Radixin–Moesin (ERM)

The plasma membrane and underlying cortical actin cytoskeleton are physically distinct entities that are interconnected by cytoskeletal proteins, such as ERM (Fehon et al. 2010; Ivetic and Ridley 2004a). Thus, dynamic changes in membrane shape, for example in microvillar formation and collapse, are structurally supported by ERM (Brown et al. 2003; Nijhara et al. 2004). Moesin is highly abundant in leukocytes, followed by ezrin and radixin is either extremely low in abundance or absent. ERM have up to 85% amino acid identity within the 3-lobed cloverleaf-shaped N-terminal domain, which contains a phosphatidylinositol 4,5-bisphosphate (PIP2)-binding domain that is proximal to a region that binds to the cytoplasmic tail of transmembrane proteins (Ivetic and Ridley 2004a). ERM adopt an autoinhibited conformation when in the cytosol, where the N- and C-termini physically interact with one another. Unfolding occurs when ERM come in contact with PIP2 and the unfolded molecule is further stabilised through phosphorylation of a conserved C-terminal threonine residue within the F-actin-binding domain (Barret et al. 2000). Taken together, the actin-binding domain and the PIP2-binding domains are critical in the membrane/cytoskeleton cross-linking activity of ERM. Ezrin possesses unique tyrosine residues at positions 145 and 353, the latter of which, when phosphorylated, supports binding of the p85 regulatory subunit of PI3-kinase (Gautreau et al. 1999). Ezrin also possesses a polyproline motif within the C-terminal half of the protein, thought to mediate protein–protein interaction. Therefore, while ezrin and moesin are highly similar, they possess unique features that could be essential for mediating non-redundant signal transduction events. Indeed, ezrin and moesin display divergent roles downstream of T-cell receptor signalling (Ilani et al. 2007).

Experiments using affinity chromatography columns, containing immobilised synthetic peptides corresponding to the tail of L-selectin, identified ezrin and moesin as novel binding partners of L-selectin (Ivetic et al. 2002). ERM were originally isolated from whole-cell extracts derived from naïve T-cells pre-treated with or without phorbol ester (phorbol myristate acetate; PMA), a potent activator of PKC and inducer of L-selectin shedding. Interestingly, ezrin from both sets of extracts could interact with the affinity column, whereas moesin could only be retained on affinity columns from extracts of cells pre-treated with PMA. Cell activation-dependent binding of moesin but not ezrin suggests that each ERM member potentially serves different roles in regulating L-selectin. Other cell adhesion molecules that mediate tethering and rolling, such as PSGL-1 and CD44, also interact with ERM. The identification of a cryptic immunoreceptor tyrosine-based activation motif (ITAM) within the N-terminal domain of ERM, suggests that ERM can act as adaptors for signal transduction events downstream of ligand engagement (Urzainqui et al. 2002). This mode of signal transduction would be particularly advantageous for cell adhesion molecules with short cytoplasmic tails, such as L-selectin. Spleen tyrosine kinase has been shown to bind to the cryptic ITAM of moesin, specifically when PSGL-1 is clustered with monoclonal antibody. It is tempting to speculate that such mechanisms occur when L-selectin is clustered but this has not been tested.

Mutagenesis of the L-selectin tail led to the discovery of a single amino acid exchange mutation that abrogates interaction of both ezrin and moesin (Ivetic et al. 2004), where arginine at position 357 is mutated to an alanine (R357A). Interestingly, cells expressing R357A L-selectin display a significant reduction in tethering efficiency under flow conditions, which could be due to the absence of the mutated protein from microvilli. R357A L-selectin is more resistant to PMA-induced shedding than WT L-selectin, implying that ERM act as pro-shedding factors. ERM are therefore paradoxically involved in anchoring L-selectin to microvilli and driving L-selectin shedding. A resolution to this paradox could be that ezrin supports L-selectin anchorage to microvilli while moesin promotes shedding. Indeed, this hypothetical view would fit with the manner in which ezrin and moesin interact with affinity columns (Ivetic et al. 2002). In silico modelling reveals that the L-selectin tail can support the simultaneous binding of CaM and ERM, leaving little space for other proteins to bind (Ivetic 2013; Killock et al. 2009). However, given that ERM and CaM can bind to other proteins, it is highly likely that higher-ordered complexes can arise from these two partners.

### Alpha-actinin and μ1A subunit of AP-1

**Fig. 2:**
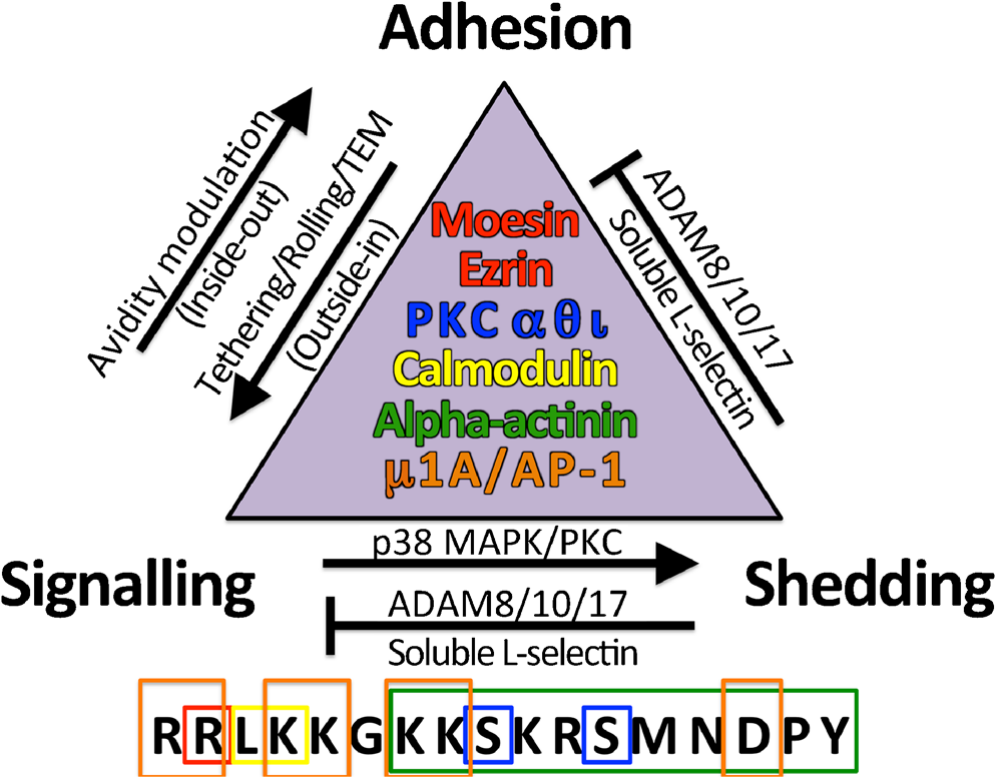
The triad of L-selectin regulation: Adhesion, Shedding and Signalling. For any given neutrophil engaged in tethering, rolling or transendothelial migration (*TEM*), L-selectin is likely to be regulated very differently in space and time. It is important to appreciate that these three aspects of L-selectin regulation are inextricably linked. L-selectin-dependent adhesion can be regulated by classic inside-out signalling, which can lead to increases in avidity modulation (for specific examples, see Table 1). Rapid shedding of L-selectin limits adhesion and signalling. Although ADAMs 8 and 10 have been shown to contribute to shedding in specific settings, ADAM17 is considered to be the dominant sheddase in neutrophils. Activation of either PKC or p38 MAPK can lead to different modes of shedding that culminate in ectodomain shedding. Moesin, ezrin, protein kinase C isozymes (*PKC*), calmodulin and alpha-actinin have all been shown to bind directly to the L-selectin tail and therefore act as direct mediators of these responses. The 17 amino acid cytoplasmic tail of human L-selectin is drawn below,* boxed and colour-matched* to their cytosolic binding partners (represented within the* triangle*)

Non-muscle alpha-actinin was the first characterised binding partner of L-selectin (Pavalko et al. 1995). Its role in cross-linking actin filaments would suggest an appropriate subcellular location in microvilli. However, alpha-actinin does not possess any known membrane/cytoskeleton cross-linking activity and so its relationship with ERM is likely to be non-redundant. The last 11 amino acids of the L-selectin tail are essential for alpha-actinin interaction, which is at the opposite end to where ERM binding is thought to occur (see Fig. 2). Cell lines expressing the 11 amino acid-truncated mutant of L-selectin revealed significantly reduced rolling efficiencies under flow conditions. Interestingly, the remaining 6 amino acids are sufficient to anchor L-selectin to microvilli (Dwir et al. 2001). Alpha-actinin plays a prominent role in integrin signalling but its role in signalling downstream of L-selectin is not clear.

μ1A of the AP-1 complex is the latest identified L-selectin binding partner and was isolated from “pull-down” experiments using extracts from Raw 264.7 mouse macrophages and the interaction validated with purified proteins (Dib et al. 2017). Pre-stimulation of Raw cells with PMA increased the affinity of μ1A for L-selectin. In contrast to all the other binding partners, the interaction of μ1A with L-selectin is thought to dominate during vesicular transport through the Golgi network. Imaging of THP-1 monocytes expressing L-selectin-GFP revealed that L-selectin/μ1A colocalised at Golgi compartments but was excluded from the plasma membrane. The N-terminal di-basic motifs and C-terminal aspartate residues in mouse L-selectin (Fig. 1a) were required for binding μ1A, which would suggest that this protein is required to occupy a substantial region of the L-selectin tail during anterograde vesicle trafficking. Phosphorylation of S364 abrogates μ1A binding. Where and when the exchange between μ1A and CaM/ERM binding occurs is unknown and will require further investigation.

### Other indirect binding partners of the L-selectin tail

Direct binding can only be confirmed unequivocally via interaction between purified proteins. Therefore, classic immunoprecipitation (IP) or pull-down procedures from whole cell extracts can at best infer indirect interaction. IP approaches have identified numerous proteins, some of which include: the Src family kinases Lyn, Hck, Fgr, DAP12 and FcRγ; c-Abl; and Grb2/SOS and Rac (referenced in Table 1). The high isoelectric point of the L-selectin tail (11.17) should be considered when performing and interpreting pull-down assays. The highly positively charged tail can give rise to false-positive binders, so pre-clearing whole-cell extracts with a scrambled form of the L-selectin tail has been proven to be highly effective in increasing the chances of isolating true binders (Ivetic et al. 2002). Complementary experimental approaches are always necessary to validate the interaction within intact cells, e.g., Förster resonance energy transfer and/or fluorescence lifetime imaging microscopy.

## Adhesion, shedding and signalling: the triad of L-selectin regulation at the plasma membrane

The past three decades of research into L-selectin has unearthed three inextricably linked aspects of regulation: adhesion, shedding and signalling. Figure 2 illustrates how each aspect of the triad is linked and lists the intracellular binding partners that interconnect them. It is important to appreciate that manipulating one aspect of the triad will invariably impact the other two aspects. Experimental design is therefore key to understanding the outcome of the result. For example, domain-swap mutations between the cytoplasmic and transmembrane domains of CD31 and CD44 with that of L-selectin have broadly addressed the importance of these domains in regulating L-selectin (Buscher et al. 2010; Fors et al. 2001). However, given the importance of cytosolic binding partners acting on disparate aspects of L-selectin function, the outcome of such results are unlikely to be clear cut. Single point mutations are therefore likely to unearth more meaningful data in respect of teasing out mechanisms associated with adhesion/shedding/signalling. The recent identification of the μ1A subunit of AP1 adaptin complex may add a fourth dimension to the current triad, delivery of translated and glycosylated L-selectin to the plasma membrane (Dib et al. 2017). However, for the purposes of this review, understanding the triad of L-selectin regulation is restricted to the mature translated and glycosylated form presented at the plasma membrane of circulating neutrophils.

## L-selectin-dependent signalling: homo/heterotypic L-selectin clustering

The scope of this review precludes a detailed description of many excellent experimental approaches that have been undertaken to understand some of the consequences downstream of L-selectin-dependent signalling. Most of what is understood about cellular behaviour downstream of L-selectin-dependent signalling stems from challenging isolated neutrophils with soluble/immobilised ligand, or with monoclonal antibody followed by clustering with a secondary antibody. While antibody-mediated cross-linking is the most guaranteed way of specifically activating L-selectin-dependent signalling, it lacks physiological relevance. In contrast, the use of glycomimetics, while more physiologically relevant, are not necessarily restricted to L-selectin-dependent adhesion and clustering (Ding et al. 2000). Such approaches have led researchers to understand that L-selectin clustering can prime or directly promote specific cellular events in neutrophils (see Table 1). Most of these include: (1) increases in intracellular calcium concentration, (2) tyrosine phosphorylation, (3) cell shape change, (4) β1 and β2 integrin activation, (5) superoxide production, (6) cell stiffening, (7) Rac activation, (8) actin polymerisation and (9) priming for degranulation. While these approaches provide extremely invaluable insight into the isolated effects of clustering L-selectin, they require further validation in more physiologically relevant models. The contribution of other signalling receptors, acting either in *cis* or *trans*, requires careful consideration when addressing the contribution of L-selectin-dependent signalling in more complex settings (for example when neutrophils are perfused over activated endothelial monolayers). Parallel plate flow chamber assays have proved particularly useful in this regard, since immobilising recombinant purified proteins (e.g., E-selectin, ICAM-1 and chemokine) allows full control over assay complexity (Morikis et al. 2017; Mueller et al. 2010; Yago et al. 2010).

The structure of the plasma membrane and its cholesterol composition (e.g., lipid rafts) are likely to play vital roles in how signals are transduced during recruitment. Heterotypic clustering of L-selectin with P-selectin glycoprotein ligand-1 (PSGL-1) has been shown to occur in neutrophils, which increases in response to E-selectin-dependent rolling. *Cis* interaction between L-selectin and PSGL-1 occurs within mutual lipid raft domains to mediate signalling to Src family kinases and trigger LFA-1 (αLβ2, CD11a/CD18) integrin activation (Stadtmann et al. 2013). Ultimately, this work postulates that L-selectin/PSGL-1 interaction might override chemokine-dependent integrin activation in a tissue-/organ-specific manner. A more recent study in human neutrophils showed that N-linked sLe^x^ presented on L-selectin binds to E-selectin under flow to drive outside–in signalling, leading to the full activation of LFA-1 and chemokine-independent arrest of neutrophils on recombinant purified ICAM-1 under flow conditions (Morikis et al. 2017). Interestingly, this mode of LFA-1 activation is independent of PSGL-1 in human neutrophils. The glycomimetic Rivipansel was used to selectively mask sLe^x^ on L-selectin over sLe^x^ moieties on PSGL-1, which exposed the contribution of L-selectin-dependent modulation of LFA-1 activity. Mouse neutrophils lack fucosyl transferase 9, which is essential for decorating N-linked glycans on L-selectin with sLe^x^. Importantly, these studies reveal fundamentally divergent signalling mechanisms between mice and humans.

## The importance of mechanosignalling in circulating neutrophils

X-ray crystallographic studies have revealed that L-selectin adopts a bent conformation in circulating neutrophils, by virtue of a hydrogen bond between tyrosine 37 (Y37) in the CTLD and asparagine 138 (N138) within the EGF-like domain (Liu et al. 2017; Mehta-D'souza et al. 2017) (Fig. 1a). Mutating N138 to glycine (N138G) in mice enabled researchers to question the in vivo significance of this hydrogen bond (Liu et al. 2017). The N138G mutation reduced the force range for transitions from catch–slip bond interactions (Fig. 1b), which increased the bond lifetime at low shear stresses and heightened priming in circulating neutrophils. As a result, circulating N138G neutrophils expressed lower surface levels of L-selectin (due to shedding) and a concomitant increase in Mac-1 expression. Compared to WT neutrophils, N138G neutrophils produced more reactive oxygen species in response to various challenges (Liu et al. 2017). While this behavioural change in N138G neutrophils improved bacterial killing compared to WT neutrophils, it also worsened outcomes in models of sterile cutaneous inflammation and venous thrombosis. Taken together, it was understood that, while in the circulation, the Y37–N138 hydrogen bond decreases the propensity for neutrophil priming. Mechanistically, the signalling mechanism is not fully understood but is known not to require PSGL-1. This observation is suggestive of L-selectin and PSGL-1 shifting into different membrane domains to mediate exclusive signalling events. The increased priming phenotype downstream of N138G L-selectin was also witnessed (but not reported) in other leukocyte subsets (Liu et al. 2017), suggesting that the phenomenon extends beyond just neutrophils. This work led to the first in vivo demonstration of “mechanochemical regulation” of L-selectin. It would be particularly interesting to understand whether human neutrophil rolling via L-selectin (sLe^x^)/E-selectin interaction has any influence on the Y37–N138 hydrogen bond and whether this contributes to neutrophil priming specifically during recruitment to inflamed endothelium.

## L-selectin: a driver of invasion and cell polarity

**Fig. 3:**
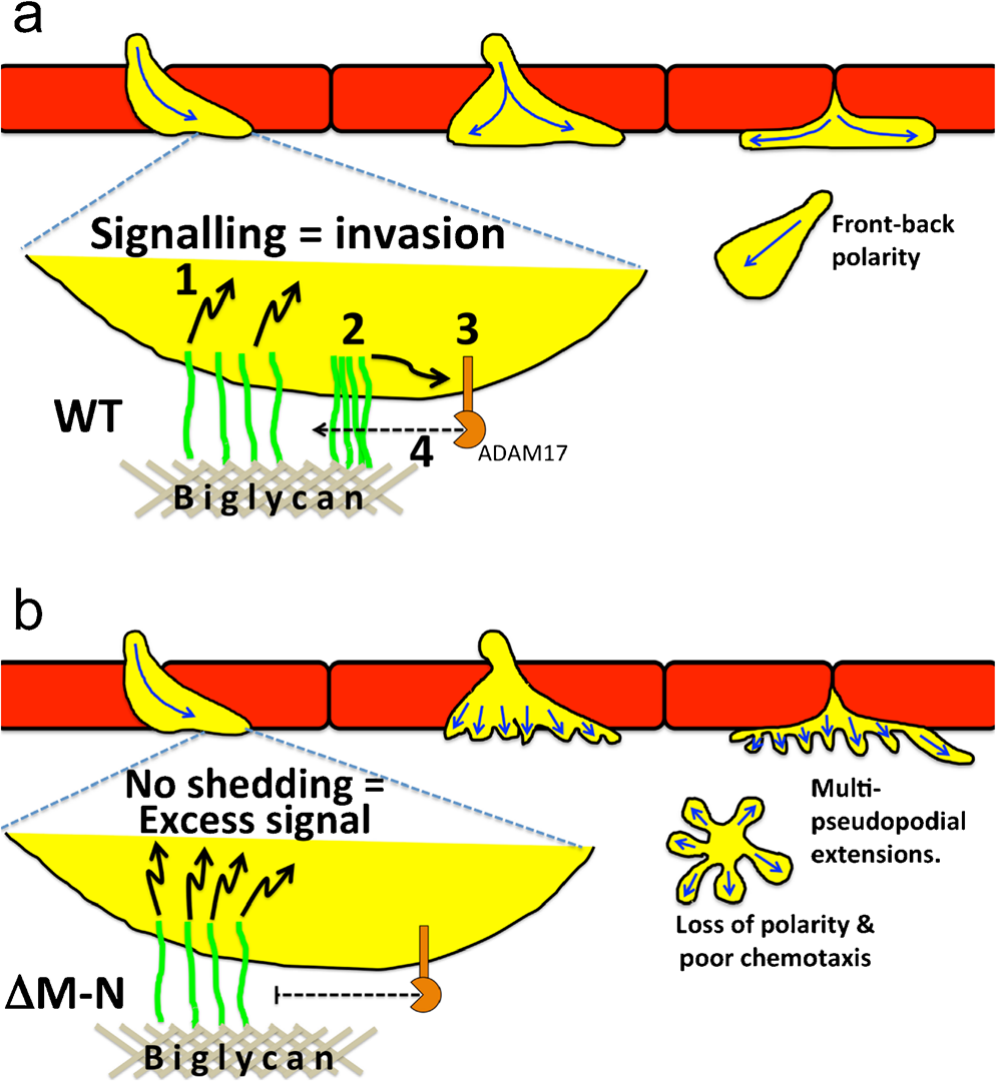
The role of L-selectin in regulating TEM and polarity in transmigrated leukocytes. **a** Neutrophils (*yellow cells*) undergoing TEM. The L-selectin (*green*) within transmigrated pseudopods makes contact with subendothelial ligands, such as biglycan, which leads to intracellular signalling (*1*). Given that L-selectin interacts with ERM proteins and that ezrin can act as an adaptor for PI3K signalling, it is hypothesised that this is the main mode of L-selectin-dependent signalling. Sustained interaction of L-selectin with biglycan leads to clustering (*2*). Signals, possibly downstream of L-selectin clustering (*2*), leads to ADAM17 activation (*3*) and subsequent ectodomain shedding of L-selectin (*4*). It is likely that other factors that are extrinsic to L-selectin clustering and signalling also play a role in this mechanism. Although the activities of PKC and p38 MAPK are known to drive L-selectin shedding (Killock et al. 2009), their exact contribution in this setting is not clear. **b** When L-selectin shedding is blocked genetically (e.g., ΔM-N) or pharmacologically, the L-selectin contacting biglycan within the subendothelial space cannot be clustered, which is thought to promote excessive signalling. ΔM-N cannot be clustered (possibly because of altered serine phosphorylation of the tail and/or altered binding to cytosolic partners), which manifests in a multi-protrusion phenotype. This in turn can profoundly affect cell polarity and persistence in directed cell migration. Further details of this work have been recently reported (Rzeniewicz et al. 2015)

L-selectin in monocytes can promote invasive behaviour specifically during TEM. Although cell lines lacking L-selectin can undergo TEM, the expression of L-selectin in such cells can significantly increase the invasion efficiency (Rzeniewicz et al. 2015). Live imaging of primary human CD14+ monocytes revealed that L-selectin is cleaved specifically during TEM and not before (Rzeniewicz et al. 2015), suggesting that full-length L-selectin can participate in signalling during TEM and before the shedding event. Indeed, confocal microscopy revealed that full-length L-selectin is present in pseudopods of monocytes captured in mid-TEM (see Fig. 3a). Mechanistically, L-selectin within transmigrating pseudopods interacts with subendothelial glycans (such as biglycan), which clusters L-selectin prior to ectodomain shedding. Moreover, as TEM proceeds, the pool of L-selectin within transmigrating pseudopods is phosphorylated at S364, leading to the dissociation of CaM and subsequent ectodomain shedding. While blocking ectodomain shedding of L-selectin did not affect overall TEM rates, cell polarity and persistence in directed cell migration of fully transmigrated monocytes was profoundly altered. Therefore, blocking the shedding of L-selectin can profoundly alter cell polarity in monocytes entering the subendothelial space (see Fig. 3b). Although WT L-selectin can cluster upon biglycan binding, the ΔM-N sheddase resistant mutant (described in “Cleavage-resistant mutants of L-selectin and sheddase inhibitors”) cannot. Blocking L-selectin shedding can therefore have profound effects on cytosolic binding partner interaction and lateral mobility along the plane of the plasma membrane. The phenotypic behaviour of monocytes in this study could help understand the in vivo observations of L(E) neutrophils emigrating from postcapillary venules in response to chemokine (Venturi et al. 2003).

## Concluding remarks

The last 30 years of research into L-selectin has unearthed some important insights into how this cell adhesion molecule regulates neutrophil behaviour. Blocking the CTLD of L-selectin has shown limited success in clinical trials (Raffler et al. 2005), which suggests that the mechanisms neutrophils employ to exit the circulation are likely to be redundant and may depend on the vascular bed in question. However, as shown recently in human monocytes, blocking the shedding of L-selectin could interfere with cell polarity and chemotaxis (Rzeniewicz et al. 2015). Given that neutrophil chemotaxis is an essential prerequisite for effector function, blocking L-selectin shedding would be worth exploring in this regard. Steering unwanted neutrophils away from sites of acute sterile injury, such as in myocardial infarction, would be an interesting avenue to explore. Lastly, targeting the sLe^x^ moiety on L-selectin as a ligand for E-selectin on circulating neutrophils (Morikis et al. 2017) could have the potential to block unwanted neutrophil recruitment in sterile injury.
